# Progress and Challenges in the Management of Congenital Cytomegalovirus Infection

**DOI:** 10.3390/clinpract14060191

**Published:** 2024-11-12

**Authors:** Weronika Szulc, Natalia Szydłowska, Julia M. Smyk, Anna Majewska

**Affiliations:** Department of Medical Microbiology, Medical University of Warsaw, Chalubinskiego 5 Str., 02-004 Warsaw, Poland; weronikaszulc00@gmail.com (W.S.); julia.lekarski@gmail.com (J.M.S.)

**Keywords:** antiviral drugs, congenital CMV infection, cytomegalovirus, foscarnet, ganciclovir, valganciclovir, vertical CMV transmission

## Abstract

Congenital cytomegalovirus (CMV) infection is the most common intrauterine viral infection with a significant impact on the foetus and newborn. Current diagnostic practice includes serological testing for specific antibodies, but there are no global screening protocols. Maternal CMV screening is often performed in conjunction with antenatal ultrasound. While most infections are asymptomatic, severe cases can lead to long-term disability or death. Antiviral therapies, mainly ganciclovir and valganciclovir, are reserved for symptomatic patients, especially those with central nervous system involvement. Although effective, these treatments are associated with significant side effects such as neutropenia and hepatotoxicity. Foscarnet and cidofovir are used as alternatives, but their efficacy and safety require further study in paediatric patient populations. The effectiveness of passive prophylaxis is still uncertain. The lack of universally accepted guidelines for diagnosis, treatment, and prevention and the risk of serious side effects highlight the need for continued research. This review evaluates current therapeutic strategies, discusses their efficacy and associated risks, and highlights the need for innovative approaches to improve outcomes for affected neonates.

## 1. Introduction

Human cytomegalovirus (CMV), also referred to as human herpesvirus 5 (HHV-5), is a member of the *Orthoherpesviridae* family and stands out as the largest herpesvirus known to infect humans [1]. Initially isolated from patients with congenital CMV infection (CCI), it derives its name from the distinctive giant cells featuring intranuclear inclusions, often described as “owl eyes” [2]. CMV infection can manifest in three forms: primary infection (in individuals with no prior exposure to CMV), secondary infection (due to the reactivation of a latent virus), and reinfection with a new strain. Following primary infection, which can be symptomatic or asymptomatic, the virus persists lifelong in a latent state, periodically reactivating, particularly in immunosuppressed individuals [3]. 

CMV infection is ubiquitous globally, with over 90% prevalence in developing countries and 40% in more developed regions with better hygiene. Its incidence increases with age, and humans serve as the sole reservoir. Transmission occurs via various bodily fluids (saliva, blood, urine, faeces, tears, semen, reproductive tract secretions, and breast milk) through droplets, sexual contact, blood, vertical routes (intrauterine, childbirth, and breastfeeding), and horizontal routes. In pregnant women, infection typically results from close contact with young children or through sexual transmission [4]. CCI is estimated to occur in five to seven out of every 1000 live births globally. Vertical transmission occurs through the placenta, the first foetal organ to be infected, after which the virus replicates in the renal tubular epithelium and targets the reticuloendothelial and central nervous systems, leading to long-term disabilities [5,6]. This type of transmission is particularly dangerous when the mother experiences a primary CMV infection during pregnancy, with the risk of CCI in the child being around 30–40%, compared to 1–2% in other cases. CCI is the most common congenital infection, affecting 0.6% of newborns [7]. There is growing evidence that both the rate of vertical transmission and the likelihood of complications vary with the gestational age at which the mother is infected, like other congenital infections like toxoplasmosis and rubella. Vertical transmission is lower in the first trimester and increases as the pregnancy progresses [8,9,10,11,12]. Conversely, early-acquired CMV infections heighten the risk of newborns developing symptoms post-birth [13,14,15]. In 90% of cases, the infection is mild or asymptomatic. However, prenatal infection can lead to foetal death or spontaneous miscarriage. If the infection is non-lethal, it can cause systemic CMV disease. 

The clinical presentation of CCI varies and can be categorized into moderate to severe congenital CMV disease (CCD), mild symptomatic CCD, and asymptomatic CCD with isolates of sensorineural hearing loss (SNHL) or with normal hearing. The categorization of congenital cytomegalovirus infection and disease is presented in Table 1 [16]. The prognosis is serious, with a mortality rate of 20–30%. Additionally, 10–15% of asymptomatic newborns with CCI may develop long-term effects such as hearing loss, impaired psychomotor development, and other neurological deficits within the first three years of life [17].

In this review, we aim to provide a thorough analysis of the current therapeutic options for CCD, critically examining their effectiveness and potential side effects. This article reviews current recommendations for the care of children with congenital CMV (cCMV) in terms of diagnosis, treatment, and the monitoring of treatment efficacy, adverse effects, and sequelae from the infection. The principles of care for children with congenital cytomegalovirus disease were defined at a symposium held during the European Society of Paediatric Infectious Diseases conference (2015), attended by clinicians from across Europe, and in the framework of the European Congenital Infection Initiative (ECCI) by the cCMV Guidelines Group under the auspices of the European Society of Clinical Virology (2023).

A systematic and comprehensive search of relevant databases, including PubMed, Scopus, Clinicaltrials.gov, the Cochrane Library, MEDLINE, and EMBASE, was conducted using keywords such as ‘congenital cytomegalovirus infection’, ‘congenital CMV infection’, ‘congenital CMV treatment’, ‘congenital CMV therapy’, ‘congenital CMV hearing loss’, ‘ganciclovir’, and ‘valganciclovir’ up until May 2024. Articles were qualified for the analysis based on their title and abstract. Only articles published in English were reviewed. A further selection was performed independently by three people, focusing on the articles’ originality and relevance to the scope of this review. The identified studies were thoroughly reviewed, and relevant data were extracted.

## 2. Laboratory Diagnosis of CMV Infection

Currently, most of the USA and EU guidelines do not recommend routine screening for CMV infection. However, CMV serological testing is offered in many countries [18]. Since it is not possible to prevent all newborns from acquiring CCI, we should diligently conduct screenings of mothers and foetuses to identify the infection at an early stage.

### 2.1. Maternal Infection

Maternal CMV infection is often asymptomatic or presents with non-specific flu-like symptoms, which makes diagnosis even more difficult. Serological testing is indicated for pregnant women with symptoms compatible with primary CMV infection (prolonged moderate fever, mononucleosis syndrome, or elevated liver transaminases). The test may also be ordered when abnormal ultrasound features suggest foetal infection (echogenic bowel, ventriculomegaly, or foetal growth restriction). According to the recommendation of a cCMV guidelines group (patronage of the European Society of Clinical Virology, 2023) CMV serology is recommended in the first trimester of pregnancy (as early as possible). Women who are negative should be retested every 4 weeks until 14–16 weeks (Grade A recommendation). Serological tests are not recommended beyond 16 weeks, except in cases with ultrasound CMV-compatible symptoms [19].

Maternal CMV infection is typically diagnosed by measuring immunoglobulin M (IgM) and G (IgG) levels or by demonstrating CMV-specific IgG seroconversion in women who were seronegative before their pregnancy. Current IgG detection tests have a high sensitivity (97–100%) and specificity (96–100%), but discordant results can occur when IgG levels are less than twice the cut-off value [19,20]. 

The presence of IgM does not confirm a recent infection, as IgM can persist in serum for more than a year after the initial infection; therefore, avidity testing should be performed. High-avidity IgG antibodies (>80%) are associated with past or secondary CMV infection. Therefore, high IgG avidity in the first trimester is highly likely to exclude maternal infection occurring during the first trimester, in the periconceptional period (±2 weeks from conception), and in the preconceptional period (2–8 weeks before conception) [19,21]. 

The risk of vertical transmission is higher in primary infection (30–40%) compared to non-primary infection (1–2%). The epidemiology of maternal non-primary infection is poorly documented [7,22]. 

Routine serological screening of all pregnant women for CMV antibodies is not recommended due to several factors: mainly, the lack of efficient maternal treatment, the difficulties in determining the prognosis of CCI, the potential negative effects of testing (anxiety or unnecessary amniocentesis), and the high CMV seroprevalence (86%) in people of reproductive age. In Europe, CMV seroprevalence in pregnant women is in the range of 50–85%, with approximately half of CMV cases resulting from primary infection [19].

### 2.2. Foetal CMV Infection 

If CCI is suspected, pregnant women are screened for foetal abnormalities via a prenatal ultrasound examination. Amniotic fluid testing for CMV DNA using a real-time polymerase chain reaction (real-time PCR) is the gold standard method. Evidence indicates that amniocentesis is most diagnostically valuable when performed at/or after 21 weeks of gestation and at least eight weeks after the maternal infection [16,23,24]. However, Enders and co-workers suggest that amniocentesis can be performed before 21 weeks of gestation (from 17 weeks). The authors found no significant difference in sensitivity whether amniocentesis was performed at ≥17 + 0 or ≥20 + 0 weeks of gestation [25]. 

Amniocentesis results have a high sensitivity (90–95%) and specificity (97–100%), ruling out foetal infection with a high certainty. A low viral load in amniotic fluid is associated with a lower risk of CCI. Cordocentesis is another diagnostic option with a similar accuracy to that of amniocentesis but presents more side effects due to the invasiveness of the procedure [16,23,24].

Recent studies are focusing on developing non-invasive, more accurate screening methods to improve the early detection and management of CCI. A group of researchers from Japan in their prospective cohort study presented a promising, non-invasive screening method that combines ultrasound findings with the PCR testing of maternal cervical secretions. They proved that ultrasound foetal abnormalities and positive PCR results in uterine cervical secretions are significant independent predictive factors for CCI in CMV IgM-positive women. This method has shown potential in accurately identifying CCIs while reducing risks to the mother and foetus [26]. This approach has not been revised and is not included in the official guidelines.

### 2.3. Diagnosis of CCI in Neonates

Universal screening for CCI in newborns is not yet a standard practice in many regions, although it is highly recommended by some doctors due to the high prevalence of asymptomatic cases. 

Suspicion of CCI in neonates is most often established on the basis of ultrasound examination and the identification of signs associated with CCD and children with confirmed SNHL. Table 2 shows the signs and symptoms in children with congenital CMV and the clinical features that require doctors to order testing for congenital CMV [26].

The virus can be present in saliva and is also excreted in urine. CMV infection in newborns should be diagnosed within the first 21 days of life, ideally as soon as possible after birth. Testing at this time makes it possible to distinguish between congenital and postnatal infections. CMV DNA should be detected by a real-time PCR in saliva (an easily obtained sample) or urine [16]. A positive CMV PCR result on a saliva specimen should be confirmed by CMV PCR on a urine specimen. CMV PCR testing on saliva has a high level of sensitivity (93–100%), a high negative predictive value (98–99%), a moderate specificity (91–99.7%), and a low positive predictive value (49–73%). False-positive results may occur due to the contamination of the sample with a virus from the genital tract or from recent breastfeeding [16,27]. 

Dried blood spots (DBSs) routinely collected in the first week after birth can be tested to detect DNA CMV in neonatal blood by PCR. DBSs are less sensitive compared to saliva or urine samples for CMV detection. This is due to the significantly lower CMV viral load in the neonatal blood than in saliva or urine, and the sensitivity of the diagnostic test’s variability (due to methodological differences between laboratories). A negative test result cannot be used to definitively exclude the diagnosis of cCMV [28]. The ECCI also recommended performing tests on DBSs for the retrospective diagnosis of cCMV, keeping in mind that the negative predictive value depends on the sensitivity of the assay (Grade A recommendation) [16].

Serological tests are of no diagnostic value in newborns. The sensitivity of the IgM test in the neonate is unacceptably low. A negative IgG result at birth in both the mother and the newborn rules out cCMV, but a positive test at birth cannot confirm or rule out cCMV. Maternal antibodies can remain in circulation for up to one year after birth. Neonatal diagnosis is generally recommended for all infants born to mothers with suspected or confirmed primary CCI during pregnancy and in any infants with suspected hearing loss at birth (Grade A recommendation). 

The development of preventive measures and effective treatment strategies for CCI is at the forefront of medical research due to their capability to diagnose CMV infection in pregnant women, diagnose CCI in the foetus and neonate, and assess the risk of transmission [29].

## 3. Management of Congenital Cytomegalovirus Infection

Research on potential treatments for CCI began 30 years ago beginning with ganciclovir and followed by valganciclovir [30]. Despite many years having passed, there are currently no licensed antiviral drugs for this infection. According to the European Expert Consensus Statement on Diagnosis and Management of Congenital Cytomegalovirus of the European Society for Paediatric Infectious Diseases (ESPID), antiviral therapy is usually not recommended in asymptomatic neonates or infants with mild symptoms, such as those with isolated intrauterine growth retardation, liver enzyme elevation, or transient thrombocytopenia. Antiviral treatment is typically reserved for congenitally infected neonates with moderately to severely symptomatic cytomegalovirus disease [16,19,31].

The treatment of newborns with CCI should only be initiated after a thorough evaluation of the potential benefits and risks, due to the harmful side effects of antiviral agents. The question remains on how to treat babies with isolated sensorineural hearing loss. Currently, there are no randomized controlled trials specifically targeting treatment outcomes in this particular group, and expert opinions on this matter are divided [16,19,30,31]. 

Two antiviral drugs are currently available for the treatment of congenital CMV infection: ganciclovir and valganciclovir.

### 3.1. Ganciclovir and Valganciclovir—Recommendation for Antiviral Therapy

Ganciclovir (GCV, 9-[(1,3-dihydroxy-2-propoxy)methyl]guanine) is a synthetic nucleoside analogue of 2′-deoxyguanosine. GCV inhibits the replication of herpesviruses, both in vivo and in vitro, and has a uniquely high potency against cytomegalovirus.

The primary mechanism of action of GCV is the inhibition of viral DNA replication. This is executed by the drug active form called ganciclovir-5′-triphosphate (GCV-TP). The transformation to the active form requires intracellular phosphorylation by three enzymes. The first of these enzymes is a viral kinase, phosphotransferase, encoded by the CMV gene UL97. This enzyme in CMV-infected cells catalyses the process of the phosphorylation of GCV to the monophosphate (GCV-MP). The monophosphate form of GCV is then converted, with the help of two cellular kinases, guanylate kinase and phosphoglycerate kinase, to the 5′-triphosphate derivative. GCV-TP competitively inhibits dATP leading to the creation of defective DNA. This occurs because GCV-TP is inserted into the DNA strand, replacing adenosine bases. As a result, phosphodiester bridges cannot be created, which results in the destabilization of the DNA strand, ultimately impairing DNA synthesis [32,33,34].

When orally administered, only 5–10% of GCV is absorbed. Since 2004, GCV has been used only parenterally. Due to its low bioavailability, it was replaced with VGCV. GCV has a less favourable safety profile compared to acyclovir (ACV), with adverse effects including hematotoxicity and nephrotoxicity [35]. If ganciclovir is administered intravenously for more than 2 weeks, a central line may be used. However, when administering it via a peripheral line, the intravenous site needs close monitoring because the extravasation of the ganciclovir solution may produce a local reaction, ulcers, and scarring [36].

Valganciclovir (VGCV) is a valine ester of GCV. It is orally administered and then metabolized into GCV by intestinal and hepatic esterases. When administered with food, VGCV has an absolute bioavailability of approximately 60% (about 10 times higher than GCV). Additionally, VGCV reaches a plasma concentration three to five times higher than GCV. VGCV is manufactured in the form of tablets and powder for oral solution [37,38]. 

Over the years, in acutely unwell neonates with severe focal or multisystem disease, the recommended first-line treatment is the administration of intravenous (IV) ganciclovir at a dose of six mg/kg/dose every 12 h for 6 weeks or oral valganciclovir at a dose of sixteen mg/kg every 12 h when the newborn is on approximately 50% enteral feeds [31,39].

A European expert consensus statement on the management of cCMV was discussed during a symposium at the European Society of Paediatric Infectious Diseases conference (2015). The experts reached a consensus and specified principles of antiviral treatment (first-line treatment, indications for treatment, and length of treatment):

- Neonates with evidence of CNS disease should receive antiviral treatment (Quality rating A, Strength of recommendation 1). Treatment should preferably be for 6 months (Quality rating B, Strength of recommendation 2);
- Neonates with no clinical or laboratory findings consistent with CMV disease should not receive treatment; there is no evidence to support treatment in this cohort (Quality D, Strength 1);
- Infants with evidence of life-threatening disease or severe single or multiple organ disease should receive treatment. The evidence is limited, especially for life-threatening disease, but there was a consensus that treatment should be considered in this group (Quality B, Strength 1). There was no consensus about the duration of treatment in this group;
- Oral valganciclovir is now the drug of choice. Intravenous ganciclovir should be used in infants who cannot tolerate oral medication or whose gastrointestinal absorption is uncertain (Quality A, Strength 1) [31].

These findings were supported by experts’ opinion from the ECCI (2023):

- Oral valganciclovir is the drug of choice. Ganciclovir can be used in infants who cannot take enteral medications or in very severe cases, with a switch to the oral route as soon as possible (Grade B);
- Antiviral treatment should be started as soon as possible and before 1 month of age (Grade A). Treatment started between 1 and 3 months of age may be beneficial. After 3 months of age, case-by-case assessment with an expert is recommended (Grade C);
- Six weeks of antiviral treatment is recommended for infants with isolated persistent hepatitis and no other manifestations of CCI at birth (grade D), and antiviral treatment is recommended for infants with isolated persistent thrombocytopenia and no other manifestations of CCI at birth (Grade D);
- Treatment for infants with isolated intrauterine growth restriction (IUGR) without other manifestations of CCI at birth is not recommended (Grade D) [19].

The internationally accepted GRADE framework was used to assess the quality of evidence. 

### 3.2. Adverse Effects of Ganciclovir and Valganciclovir 

Although both GCV and VGCV are currently considered for the treatment of CCD, their use is unfortunately associated with some side effects. The most common ones are as follows:

- Neutropenia, which is the primary adverse effect of intravenous GCV. It has been observed in 25% to 60% of infants who received this antiviral drug. It also occurs with VGCV use, although less commonly, affecting approximately 20% of these patients. Fortunately, severe cases are not common, and most often, neutropenia resolves when withholding treatment for 1 to 7 days. After this period, it is usually possible to restart the therapy without reducing the dose;
- Thrombocytopenia, which occurs in 6% of babies who received intravenous GCV. However, it is common for children born with CCI to have a low platelet count at birth. In a randomized controlled trial, Kimberlin et al. reported that there was a similar incidence of low platelet counts (<50,000/mm^3^) in newborns treated with intravenous GCV and in untreated infants (7% vs. 5%, respectively);
- Nephrotoxicity: an increase in serum creatinine concentration was reported in less than 1% of infants;
- Hepatotoxicity: children treated with IV GCV, especially at a dose of 6 mg/kg or higher, may experience elevated levels of hepatic transaminase;
- Local inflammatory reactions, ulcers, and scars can be caused by the extravasation of the drug during GCV treatment [30,34,40].

Antiviral treatment therefore needs to be monitored. It is recommended that a complete blood count and a liver function test be carried out regularly during antiviral treatment (Grade B). Checking the full blood count and liver function test regularly during antiviral treatment is recommended (Grade B) [19,31].

Ganciclovir has been associated with both gonadotoxicity and carcinogenicity, but most evidence comes from animal studies rather than extensive human trials. In preclinical studies involving mice, ganciclovir was shown to cause tumours in various organs, including the preputial and clitoral glands, forestomach, ovaries, uterus, and liver. While these findings raised concerns about its carcinogenic potential in humans, it is important to note that some of the affected tissues in animals, such as the preputial and clitoral glands, do not have direct human counterparts. Despite this, ganciclovir is still considered a potential carcinogen in humans based on these results.

Regarding gonadotoxicity, studies on animals demonstrated that ganciclovir could lead to reduced fertility and testicular atrophy, with male mice and dogs showing decreased spermatogenesis. In female mice, decreased fertility and ovarian atrophy were observed. These effects were dose-dependent, with some being reversible at lower doses but irreversible at higher doses. However, no direct human clinical studies have conclusively confirmed these toxicities, and data from follow-up studies on children treated with ganciclovir are still under review for its long-term effects on sexual development and cancer incidence.

For clinical decisions, the use of ganciclovir is often carefully weighed against its benefits, particularly in severe conditions like CMV infections, but its toxicological risks remain a consideration in long-term or high-dose treatments [41,42].

### 3.3. An Overview of Research Supporting Antiviral Treatment 

In a randomized, controlled study published by Kimberlin et al., 100 patients were enrolled. Newborns with symptomatic CCI involving the CNS were randomly assigned to receive either 6 weeks of intravenous ganciclovir or no treatment. Among those who received ganciclovir, 84% showed improved hearing or maintained normal hearing levels at the 6-month follow-up brainstem-evoked response (BSER) examination. In contrast, only 59% of patients in the control group exhibited similar results. None of the ganciclovir recipients experienced a worsening of hearing, whereas 41% of untreated patients showed deteriorated BSER results. After a year or more, 21% of ganciclovir-treated patients experienced hearing deterioration, compared to 68% in the control group. Ganciclovir appeared to be a promising option for treating newborns with symptomatic CCI involving the central nervous system, as it helped prevent hearing deterioration at 6 months and offered continued protection beyond one year. Regarding the safety of ganciclovir, this study, like many others, highlights the issue of neutropenia during antiviral treatment. Patients treated with ganciclovir were significantly more likely to experience severe neutropenia (Grade 3 or 4), with 63% of the study group affected, compared to 21% in the control group [43]. 

In a similar study by Whitley et al., hearing improvement or stabilization 6 months after ganciclovir treatment occurred in 16% of patients. As for safety, 88% of the patients developed thrombocytopenia while 69% developed total neutropenia [44].

In a case series by Michaels et al., nine cases of children with symptomatic CCI were presented. All were treated with intravenous ganciclovir for a median duration of one year, followed by oral ganciclovir for a median duration of ten months. Prior to therapy, hearing loss was observed in five patients. After a median follow-up of two years, no child experienced a progression of hearing loss, and two children showed improvement in their hearing. Seven children encountered complications from the treatment, primarily related to the intravenous administration of the drug, including central venous catheter infections and catheter malfunctions [45].

What is more, ganciclovir can potentially be effective not only in the treatment of hearing impairments but also in the treatment of chorioretinitis in newborns with CCI. In the case report published in 2010, Shoji et al. described a male newborn with confirmed CCI, born at 38 weeks of gestation and successfully treated with ganciclovir [46].

In a 2015 randomized, placebo-controlled clinical trial, Kimberlin et al. evaluated the efficacy of valganciclovir for treating CCI. The trial included 96 neonates who initially received valganciclovir at a dose of 16 mg per kilogram of body weight, taken orally twice daily, for 6 weeks. Following this period, the participants were randomized to either continue valganciclovir or switch to a placebo for an additional 4.5 months, with the dosage adjusted monthly according to body weight. The study concluded that extending valganciclovir treatment to 6 months did not enhance short-term hearing outcomes compared to the 6-week regimen. However, sustained benefits were observed at 12 and 24 months, with the 6-month treatment group showing significantly better neurodevelopmental outcomes at 24 months [47].

Similarly, the study conducted by Amir et al. provides further evidence of the benefits of prolonged antiviral therapy for CCI. This retrospective analysis reviewed the medical records of 23 infants with CCI and CNS involvement. The treatment protocol involved administering intravenous ganciclovir at 5 mg/kg every 12 h for 6 weeks, followed by oral valganciclovir for up to 12 months. Initially, the infants received two daily doses of valganciclovir every 12 h for the first 6 weeks, after which they continued with a single daily dose, with the dose adjusted for each kilogram of body weight. The study found that by the age of one year or older, 76% of the affected ears had normal hearing, an improvement from 54% at baseline. Additionally, viral load monitoring indicated a sustained virological response throughout the treatment period. The authors concluded that prolonged therapy, starting with intravenous ganciclovir and followed by oral valganciclovir, appeared to result in better auditory outcomes than shorter-term treatments. In terms of safety, the main side effect of treatment was as often described in other papers—transient neutropenia [48].

In 2017, Seidel et al. conducted a systematic review of case reports detailing the use of ganciclovir or valganciclovir during pregnancy for managing foetal or maternal CMV infection. Four of the reviewed cases involved symptomatic CMV infections in pregnant women and discussed the potential side effects of these treatments on the foetus. The authors concluded that, despite possible embryotoxic effects and potential impacts on germ cells, there are no documented cases of malformations resulting from in utero exposure to ganciclovir or valganciclovir. However, these cases exhibit significant heterogeneity, and none report long-term outcomes. The review also included three cases in which ganciclovir or valganciclovir was administered to pregnant women with the intention of treating the foetus following confirmed foetal infection. In one case study, intrauterine ganciclovir was administered via cordocentesis to the foetus, which was associated with preterm stillbirth. Furthermore, two cases involved oral valganciclovir treatment during pregnancy. In both cases, oral valganciclovir therapy at a dosage of 450 mg three times a day was initiated. No side effects were reported in the mothers. Post-delivery, the children exhibited no obvious malformations, and hearing tests revealed no abnormalities. DNA CMV was detected in the urine of the newborns at 10 and 15 days of life, respectively, and valganciclovir treatment was subsequently started. At the ages of 3 months and 5 months, neither hearing loss nor neurological abnormalities were detected in the children [49].

In addition, in 2011, Kashiwagi et al. presented a case report on the long-term treatment of a newborn with symptomatic CMV infection using valganciclovir. An automated auditory brainstem response test conducted at 5 days old revealed severe hearing impairment, and cranial magnetic resonance imaging (MRI) at 11 days old showed abnormal findings. Valganciclovir treatment commenced at 5 weeks of age and was continued for 6 weeks. Due to the improvement in hearing and the absence of adverse effects, the treatment was extended for an additional 6 weeks. Over the course of 6 months, neither the hearing impairment nor the cranial MR imaging results worsened [50].

In a study conducted by Kimberlin et al. that enrolled neonates with symptomatic congenital CCD, the pharmacokinetics of a valganciclovir oral solution and intravenous ganciclovir were evaluated. They found that a dose of valganciclovir (16 mg/kg/dose) every 12 h resulted in a plasma concentration of ganciclovir similar to that achieved with intravenous ganciclovir. The study also indicated that the use of an oral antiviral posed a lower risk of developing neutropenia compared to intravenous ganciclovir [40].

A retrospective study conducted by Bilavsky et al. has shown that babies with CCI and hearing impairment who received antiviral treatment for 12 months displayed a significant improvement in their auditory outcome [51]. However, further research is needed to establish the optimal treatment duration.

In seven other studies, a reduction in the number of children with SNHL after therapy (IV GCV followed by oral VGC or oral VGC followed by IV GCV) was observed [52,53,54,55,56,57,58]. 

In a randomized, double-blind, placebo-controlled phase II trial (ClinicalTrials.gov identifier NCT01649869), 54 participants were enrolled (35 with cCMV-associated SNHL, with 17/18 receiving antiviral therapy/a placebo). The authors aimed to determine if valganciclovir initiated after 1 month of age improves SNHL associated with cCMV. This trial did not confirm that six weeks of oral valganciclovir therapy improves audiological outcomes six months after treatment initiation in children with CCI-associated SNHL [59].

In five other studies, all of which were case reports, the number of children with SNHL after therapy remained unchanged [60,61,62,63,64].

## 4. Monitoring

All infants diagnosed with congenital CMV infection should be monitored and followed up, regardless of whether symptoms are apparent at birth. Audiology, ophthalmology, and neurodevelopmental follow-ups should be conducted for all children, irrespective of whether they receive antiviral treatment [16,19].

Recommendations for the monitoring of children undergoing antiviral treatment for CCI, supported by the results of several research papers, can be found in the aforementioned European Society of Paediatric Infectious Diseases (ESPID) guidelines and the ECCI’s guidelines [19,31].

GCV or VGCV treatment requires regular haematological monitoring due to the risk of myelosuppression, including neutropenia and thrombocytopenia. The neutrophil and platelet count needs to be checked weekly during the initial 6 weeks of treatment, then at 8 weeks, and once a month afterwards for the rest of the therapy. Additionally, liver (aspartate aminotransferase, alanine aminotransferase, and total and direct bilirubin levels) and renal function tests (blood urea nitrogen and creatinine levels) should be performed weekly in the first month and then at least monthly (Quality B, Strength 2) [19,40,47]. To evaluate the effectiveness of antiviral treatment, it is useful to check the viral load (quantitative DNA CMV PCR measurements of whole blood or plasma) (Quality C, Strength 2). No agreement has been reached on the frequency of viral load monitoring (Quality D, strength 2) [19,36,65,66].

The recommendations for paediatric infectious diseases and general paediatrics state that children with CCI and confirmed transmission in the first trimester or transmission with unknown timing should be followed-up with from birth, throughout treatment, at 6 and 12 months of age, and then annually until school age (Grade D). Asymptomatic children with normal imaging and documented maternal primary infection in the second or third trimester can receive standard paediatric care (Grade A). Vestibular screening tests should be performed within the first year of life in high-risk children (those with maternal infection in the first trimester, maternal infection of unknown timing, hearing loss, or developmental delay) (Grade B) [19]. 

The ECCI also recommend that children with clinical symptoms at birth and/or evidence of long-term sequelae such as neurological disease, SNHL, chorioretinitis, and/or neurodevelopmental impairment should be followed-up with at least annually until the age of 6 years to ensure specialized management (Grade A) [19].

Ophthalmological follow-up is recommended annually, at least until the children can speak, for those with clinically detectable disease at birth (quality C, strength 1) [31]. According to the ECCI, ophthalmological follow-up is only recommended for those infants with retinitis at birth and is not required for newborns with a normal retinal examination (Grade B) [19].

Moreover, children being treated for CCI should have hearing examinations every 3–6 months in the first year of life, then every 6 months until 3 years of age, and then every year until they turn 6 years old (Quality C, Strength 1) [31]. In cases of hearing loss at birth, regular hearing testing for as long as required is recommended (Grade A) [31].

In infants with normal hearing at birth, with a CMV infection of unknown timing during pregnancy or with a known first-trimester infection, the ECCI recommends hearing follow-ups until at least 5 years of age (Grade A). The estimated risk of delayed SNHL in asymptomatic infants without SNHL at birth and with a proven maternal primary CCI in the second trimester is low, and there is not a full consensus on whether these cases need hearing follow-ups (Grade D). In children with a proven maternal primary CCI in the third trimester and normal hearing at birth, they do not recommend hearing follow-ups (Grade A) [31].

A neurodevelopmental assessment should be conducted at 1 year of age for every child undergoing antiviral treatment for CCI. Experts recommend evaluation by a paediatric neurologist for all children with neurological symptoms and/or significant neuroimaging findings and for children with neurological problems that arise during follow-up (Grade A). Neurodevelopmental assessment is recommended at 24–36 months of age in high-risk children, as well as further follow-ups and interventions, according to findings (Grade D) [31].

## 5. An Alternative and Supportive Therapy 

While VGCV and GCV are the preferred antivirals, they are not suitable for every child. A group of patients with GCV resistance, GCV toxicity requires an alternative therapy. Fortunately, there are two drugs, foscarnet and cidofovir, which may be an option if the initial treatment fails [30].

### 5.1. Antiviral Drugs

**Foscarnet**

Foscarnet (FOS; Na_3_CO_5_P × 6 H_2_O) is a trisodium salt of phosphonomethanoic acid, and it is the only drug for herpes infection that is not a nucleoside analogue. It acts by inhibiting the activity of viral DNA polymerase, binding selectively and reversibly to its site. As a result, it prevents the DNA polymerase from cleaving pyrophosphate from the terminal nucleoside triphosphate added to the growing DNA chain. FOS was approved for the treatment of infections caused by cytomegalovirus in 1991 [67].

FOS can be used in patients with GCV and VGCV resistance as it does not have to undergo phosphorylation by kinases. This means it is effective against pUL97 kinase variations, which confer GCV and VGCV resistance. However, despite FOS’s many benefits, a significant concern arises due to the lack of research on its pharmacokinetics and safety in the paediatric population [34,68].

Important limitations of FOS therapy include significant adverse effects such as nephrotoxicity (e.g., interstitial nephritis, acute renal tubular necrosis, and electrolyte derangement), although it seems that FOS causes minimal myelosuppression [35].

The first mention of FOS’s effectiveness and safety in treating infants with CCI was described in a case report by Nigro et al. (2004). The report described a case of a boy born at a gestational age of 41 weeks. Prenatal ultrasonography showed hepatomegaly with ascites with CMV DNA (36 000 copies/mL) detected in the amniotic fluid. The baby was born with hepatomegaly, jaundice, interstitial pneumonia, hyperechoic lesions in the basal ganglia, and right microphthalmia with a cataract. He had a liver biopsy, which detected DNA CMV. The parents refused GCV therapy due to possible side effects associated with it. Therefore, the infant received FOS at a dose of 60 mg/kg every 8 h for three weeks, followed by 100 mg/kg three times a week for three months. In the course of the 3-month treatment, his chest X-ray and brain ultrasound results normalized. Additionally, the liver biopsy was repeated after five months, and it was negative for DNA CMV detection. A 10-year follow-up showed normal liver function and neurological development [69].

Another case report describing the use of FOS in the treatment of cCMV infection was reported by Knorr et al. (2007). This one involved a child who was delivered by caesarean section at 24 weeks gestation, weighing 700 g. At 8 days old, the infant presented signs of septicemia and had an elevated C-reactive protein (CRP) level. In the following days, the increase in CRP continued, and at 15 weeks, CMV IgG and IgM antibodies were detected in serum. Additionally, DNA CMV was detected by PCR in the patient’s peripheral blood leucocytes and urine. The patient was treated with granulocyte colony-stimulating factor (GCSF) and ganciclovir, which after 3 weeks of therapy, did not have the desired effect. The child was then diagnosed with CMV-associated hemophagocytic lymphohistiocytosis and started on treatment with FOS at the dosage of 100 mg/kg/day and methylprednisolone at the dosage of 2 mg/kg/day. Throughout the 3-week treatment and at an 18-month follow-up, the patient’s neutrophil and platelet counts remained normal, and their physical and neurological development showed no abnormalities [70].

Both case reports did not mention any adverse effects related to FOS use. However, the most common side effects associated with this medication are nausea, electrolyte derangements (hypocalcaemia and hypomagnesemia), and impaired renal function. Less commonly, FOS can cause seizures [68]. Resistance to FOS is associated with mutations at the pyrophosphate binding site of viral DNA polymerase [67].

The data on FOS use and its efficacy in the treatment of CCI infection remain limited.

**Cidofovir**

Cidofovir (CDV; 1-[(S)-3-hydroxy-2-(phosphonomethoxy)-propyl]cytosine dihydrate) is a monophosphate cytidine nucleotide analogue. It dramatically suppresses CMV replication by competitively inhibiting the incorporation of the host’s deoxycytidine into the viral DNA. To convert into its active form, CDV goes through a process in which it is first phosphorylated to its monophosphate metabolite and then to CDV diphosphate. This metabolite not only inhibits CMV polymerases but also acts as an alternative substrate for viral DNA polymerase, competing with the natural substrate deoxycytidine triphosphate. Once CDV is incorporated into the growing viral DNA chain, it significantly reduces the rate of viral DNA synthesis. Unlike the phosphorylation process of GCV or VGCV, the phosphorylation of CDV does not rely on viral enzymes. Therefore, it can be used in treating patients infected with CMV isolates with the UL97 mutation [34,71,72]. 

The main disadvantage that comes with the use of CDV is that it can cause renal impairment. The concentration of CDV in kidney cells is 100 times higher than in other tissues, which explains the drug-related proximal tubular injury manifested by proteinuria and glycosuria [35]. There are no studies on the pediatric population, however, this side effect was reported by clinical trials in approximately 50% of adults [34]. The drug is available in a form requiring intravenous infusions [35].

### 5.2. Hyperimmune Globulin

The use of hyperimmune globulin (HIG) in the treatment of CCD was researched by Hughes et al. They conducted a multicentre, double-blind trial involving 399 pregnant women with primary CCI infection, diagnosed before 24 weeks of gestation (ClinicalTrials.gov number NCT01376778). The participants were randomly assigned either a monthly infusion of CMV HIG (at a dose of 100 mg per kilogram of body weight) or a matching placebo until their delivery. The study showed no benefit associated with HIG treatment in terms of reducing the incidence of congenital CMV infection or foetal or neonatal death [73]. In a 2-year follow-up involving the children of the women participating in the trial, death or CCI with severe disability was reported in 13.4% of the children in the group receiving the HIG and in 10.1% of the children in the placebo group. In this trial, HIG did not improve the patients’ 2-year hearing or developmental outcomes [74].

While some studies affirm the efficacy and safety of using HIG for treating and preventing congenital CMV infection [75,76,77,78], several retrospective observational studies do not support its positive impact in treating CCI [75,76,77,79,80,81,82,83]. This highlights a need for further research to clarify the role of HIG in CCI management.

### 5.3. Supportive Treatment

Children with severe cases of CCDI may require supportive care in addition to antiviral therapy. The specific care needed varies depending on the individual patients, but may include fluid therapy, respiratory support, nutritional support, and blood transfusions [30].

## 6. Prophylaxis and Preventive Strategies

Preventing CCI involves a multi-faceted approach combining personal hygiene practices, education, screening, and ongoing research into antiviral treatments and vaccines.

### 6.1. Valaciclovir

The effectiveness of prenatal valaciclovir (VACV) administration in pregnant women with CMV infection was evaluated by D’Antonio et al. in a meta-analysis from 2023. The analysis included eight studies from the MEDLINE, EMBASE, and Cochrane databases, involving a total of 620 women. The study concluded that administering oral VACV during pregnancy significantly reduces the risk of CCI in newborns. Specifically, the risk of CCI was significantly lower in newborns whose mothers received VACV compared to those who did not. Furthermore, the analysis revealed a notably lower risk of vertical transmission in cases in which VACV was administered following a first-trimester maternal infection. Conversely, there was no significant difference in CCI risk between the newborns of the mothers who contracted CMV in the periconceptional period or during the third trimester. Additionally, the children of the mothers treated with VACV were more likely to have an asymptomatic CCI compared to those whose mothers did not receive the drug. Despite these promising findings, the quality of evidence was assessed as very low according to the GRADE system [84].

Additionally, in their retrospective, multicentre study, Egloff et al. (2023) also confirmed that VAC reduces the risk of the maternal–foetal transmission of CMV. The study involved 143 women, who were given eight g of oral VACV daily. Furthermore, researchers evaluated the nature and frequency of maternal adverse effects associated with valaciclovir treatment. The adverse effects observed were generally mild and non-specific. Back pain was reported by 6.8% of the women in the study, while 5% experienced gastrointestinal disturbances and 5% reported nausea. Dizziness and macrocytosis were reported by one patient each. One patient developed acute renal failure associated with back pain and pruritus 14 days after starting the valaciclovir treatment. The patient had no relevant past medical history. Their renal function normalized spontaneously within a few days after discontinuing the treatment. The pregnancy proceeded without further complications, and the patient delivered a healthy, asymptomatic, uninfected child at term [85].

In the same year, Chatzakis et al. presented the results of a meta-analysis on the effectiveness of valaciclovir as a secondary prevention of CCI, following primary maternal infection acquired periconceptionally or in the first trimester of pregnancy. This meta-analysis included three studies involving 527 women. The analysis demonstrated that oral valaciclovir, administered at a dosage of eight g per day, significantly reduced the rates of the vertical transmission of CMV during both the periconceptional period and the first trimester. Moreover, valaciclovir markedly lowered the incidence of CCI in newborns. The outcomes were positively correlated with the gestational age at the initiation of treatment, underscoring the importance of timely interventions. These findings further support the therapeutic potential of valaciclovir in reducing the risk of CCI and its associated complications in newborns [86].

Another study highlighting the effectiveness of valaciclovir in reducing the rate of foetal CMV infection following maternal primary infection early in pregnancy was a prospective, randomized, double-blind, placebo-controlled trial conducted by Shahar-Nissan et al. This study included 90 women infected with CMV periconceptionally or in the first trimester, with half receiving oral valaciclovir at a dosage of eight g per day, taken twice daily. The results showed that amniocentesis was significantly less likely to be positive for CMV in the valaciclovir group compared to the placebo group (11% vs. 30%). Additionally, when focusing on patients infected with CMV during the first trimester, the difference was even more pronounced (11% in the valaciclovir group vs. 48% in the placebo group). These findings demonstrate that early treatment of pregnant women with primary CMV infection using valaciclovir can significantly reduce the risk of CCI and its associated complications in newborns [87].

Valaciclovir has also shown promise in the in utero treatment of CCI, as demonstrated in a multicentre, open-label phase II study by Leruez-Ville et al. This study involved 43 pregnant women carrying foetuses with moderate symptoms of CMV infection, who were administered a high dose of 8 g of oral valaciclovir daily. The results were notable: the proportion of newborns born without symptoms increased significantly from 43% in the untreated group to 82% in the treated group. These findings suggest that valaciclovir not only reduces the risk of vertical CMV transmission and CCI but also substantially improves the likelihood of delivering a healthy newborn [88].

### 6.2. Primary Prevention

The development of preventive strategies is crucial for reducing the risk of CMV infection during pregnancy.

Firstly, to avoid CCIs in newborns, their pregnant mothers should follow strict hygiene practices. These include regular and thorough hand washing with soap and water, especially after changing their older children’s diapers, feeding them, wiping their nose, or handling their toys. Alcohol-based hand sanitizers are also recommended. Sharing food, drinks, utensils, and toothbrushes with young children is strictly forbidden, along with kissing children on the lips. It is important to underline that the risk of becoming infected with CMV for the first time is higher for pregnant women who live with young children or work in childcare settings. Those vulnerable groups should strictly follow preventive recommendations [89,90]. Awareness among pregnant women is crucial to prevent CMV infection in newborns. As healthcare providers, it is our responsibility to educate expectant mothers about the virus and its transmission.

While no CMV vaccine is currently available, its development remains a high priority for reducing congenital CMV infections in the future. According to clinicaltrials.gov, multiple phase I and phase II trials have been conducted, and there is significant interest in creating a vaccine that could be administered to women of childbearing age and their older children to prevent CMV transmission and CMIs. Moderna’s mRNA-1647 vaccine is a promising candidate and is currently in phase III of a clinical trial named CMVictory. The study involves 7454 women of childbearing age (16–40 years of age) who are in close contact with at least one child 5 years of age or younger for at least 8 h a week and who are aged 20 or older. The study is supposed to end in April 2026 [91,92].

## 7. Conclusions

In conclusion, there is an urgent need to develop new preventive strategies and treatment options for CCIs. CMV poses a significant threat to neonatal health, often resulting in long-term disabilities and developmental issues. Current interventions are limited and not universally effective, underscoring the necessity for innovative research and development in this area. By prioritizing the advancement of vaccines, antiviral therapies, and comprehensive screening programs, we can significantly reduce the incidence and impact of CCIs. Investing in these efforts not only has the potential to improve the quality of life for affected newborns and their families but also to alleviate the broader public health burden associated with this infection.

This review is an attempt to outline the diagnostic tests that can be ordered to confirm suspected congenital CMV infection and the benefits and risks of antiviral treatment. This review is not a complete compilation of expert recommendations, which is a limitation of this work and a limitation for the care of children with congenital CMV disease. It is a collection of options, both diagnostic and therapeutic, with proven efficacy in specific conditions, sometimes studied in limited numbers of patients. This summary should therefore be considered as a guide to help clinicians make clinical decisions.

## Figures and Tables

**Table 1:** Definitions of congenital cytomegalovirus infection and disease.

Moderate to severe symptomatic CCD	Mild symptomatic CCD
▪ Manifestations attributable to CCI: thrombocytopenia, petechiae, hepatomegaly, splenomegaly, intrauterine growth restriction, and hepatitis (raised transaminases or bilirubin), or ▪ CNS involvement such as microcephaly, radiographic abnormalities consistent with cytomegalovirus CNS disease (ventriculomegaly, intracerebral calcifications, periventricular echogenicity, and cortical or cerebellar malformations), abnormal CF indices for age, SNHL, chorioretinitis, or the detection of cytomegalovirus DNA in CF	One or two isolated manifestations of CCI that are mild and transient (e.g., mild hepatomegaly or a single measurement of low platelet count or raised levels of alanine aminotransferase).
Asymptomatic CCD with isolated SNHL	Asymptomatic CCD
No apparent abnormalities to suggest CCD, but SNHL is present (≥21 decibels)	No apparent abnormalities to suggest CCD; normal hearing

Legend: CCI; congenital cytomegalovirus infection, CCD; congenital cytomegalovirus disease, CF; cerebrospinal fluid, CNS; central nervous system, SNHL; sensorineural hearing loss.

**Table 2:** Possible signs and symptoms in children with congenital CMV and clinical features that require doctors to order testing for congenital CMV.

**Physical Examination** Small for gestational age (birth weight <−2 SD for gestational age) Petechiae or purpura (usually found within hours of birth and persist for several weeks) Blueberry muffin rash (intra dermal hematopoiesis) Jaundice ^1^ Hepatomegaly, splenomegaly **Neurologic physical examination** Microcephaly (head circumference <−2 SD for gestational age) ^2^ Neurologic signs (lethargy, hypotonia, seizures, poor sucking reflex) Abnormalities detected incidentally or through subsequent investigation/specialist examination **Laboratory results** Anemia Thrombocytopenia (occurs in the 1st week but platelets often increase spontaneously after the 2nd week) ^3^ Leukopenia, isolated neutropenia Elevated liver enzymes (ALT/AST) Conjugated hyperbilirubinemia Cerebrospinal fluid; abnormal cerebral fluid indices, positive CMV DNA **Neuroimaging** Calcifications, periventricular cysts, ventricular dilatation, subependymal pseudocysts, germinolytic cysts, white matter abnormalities, cortical atrophy, migration disorders, cerebellar hypoplasia, lenticulostriatal vasculopathy ^4^ **Hearing test** Sensorineural hearing loss uni- or bilaterally **Visual examination** Chorioretinitis, cataracts Retinal hemorrhage, optic atrophy, strabismus
**Other indications:** Maternal serology: Evidence of maternal seroconversion (considered in women with known CMV infection who are known to be IgG-seropositive at start of pregnancy), particularly, if symptoms or virologic examination are consistent with suspected CMV reactivation/reinfection Prematurity (baseline screening to differentiate between congenital and postnatal CMV infection is helpful for extremely premature infants) Older children: Sensorineural hearing loss: new diagnosis **Complementary information on indications for diagnosis of congenital infection** ^1.^ Prolonged jaundice with transaminitis ^2.^ Considered if symmetrically small for gestational age ^3.^ Unexplained thrombocytopenia, considered if leucopenia or anemia is present ^4.^ Considered in the case of periventricular cysts, subependymal pseudocysts, germinolytic cysts, white matter abnormalities, cortical atrophy, migration disorders, cerebellar hypoplasia, or lenticulostriate vasculopathy
